# General Practitioners’ Mental Well-Being During Crises: Results of the PRICOV-19 Study Pilot in Serbia

**DOI:** 10.3390/healthcare13050573

**Published:** 2025-03-06

**Authors:** Milena Santric Milicevic, Katica Tripkovic, Nenad Bjelica, Milan Dinic, Danilo Jeremic, Esther Van Poel, Sara Willems, Zoran Bukumiric

**Affiliations:** 1 Institute of Social Medicine, Faculty of Medicine, University of Belgrade, Dr Subotica 15, 11000 Belgrade, Serbia; milena.santric-milicevic@med.bg.ac.rs; 2 Laboratory for Strengthening Capacity and Performance of Health System and Workforce for Health Equity, Faculty of Medicine, University of Belgrade, Dr Subotica 15, 11000 Belgrade, Serbia; milandinic@yahoo.com (M.D.); drdanilo1987@gmail.com (D.J.); 3 City Institute of Public Health Belgrade, Bulevar Despota Stefana 54a, 11000 Belgrade, Serbia; katica.tripkovic@zdravlje.org.rs; 4 Clinic for Psychiatric Diseases “Dr. Laza Lazarevic”, Višegradska 26, 11000 Belgrade, Serbia; n.bjelica74@gmail.com; 5 Serbian Medical Chamber, Kraljice Natalije 1–3, 11102 Belgrade, Serbia; 6 Institute for Orthopaedics “Banjica”, Faculty of Medicine, University of Belgrade, Mihajla Avramovića 28, 11000 Belgrade, Serbia; 7 Equity in Healthcare Research Unit, Department of Public Health and Primary Care, Ghent University, C Heymanslaan 10, 9000 Ghent, Belgium; esther.vanpoel@ugent.be (E.V.P.); sara.willems@ugent.be (S.W.); 8 Quality and Safety Ghent, Department of Public Health and Primary Care, Ghent University, C Heymanslaan 10, 9000 Ghent, Belgium; 9 Institute for Medical Statistics and Informatics, Faculty of Medicine, University of Belgrade, Dr Subotica 15, 11000 Belgrade, Serbia

**Keywords:** general practitioners, mental well-being, primary health care, crisis interventions, Mayo Clinic Mental Well-Being Index, Depression Anxiety Stress Scales-21, data correlations, linear regressions

## Abstract

Background/Aims: This study was conducted with the aim of assessing the mental well-being of general practitioners (GPs) amidst the COVID-19 pandemic in Serbia. These findings are intended to provide valuable insights to primary care stakeholders about the potential need for support interventions. Materials and Methods: In the context of the international cross-sectional survey on primary health care during the COVID-19 pandemic (PRICOV-19), our initial focus was on evaluating the appropriateness of employing the Mayo Clinic Well-Being Index (MWBI) for Serbian GPs. The Spearman test validated the correlation between the GPs’ scores of the MWBI and Depression Anxiety Stress Scales-21 (DASS21) in the Serbian context. The univariate and multivariate linear regressions modeled the personal and job-related potential predictors of higher MWBI scores (*p* < 0.05). Results: A strong, positive, and significant correlation was found between the MWBI score; the total DASS21 score; and the scores for depression, anxiety, and stress (*p* < 0.001). In this pilot study, 71.3% of the GP respondents had poor mental well-being indicated with MWBI scores ≥ 2 (the mean was 3.3 ± 2.7). The likelihood of experiencing poor mental well-being among the GPs was found to be associated with decreases in their socioeconomic statuses (B = −0.893; *p* = 0.021). Furthermore, inadequate allocation of time for the review of scientific evidence and guidelines has been correlated with a decline in mental well-being among respondents (B = −1.137; *p* = 0.033). Conclusions: The MWBI effectively assessed GPs’ mental well-being amidst COVID-19 in Serbia. GPs with low socioeconomic statuses might most benefit from mental well-being support during crises. For better mental well-being, GPs need adequate time in their agendas to assess scientific evidence and adhere to established guidelines.

## 1. Introduction

Mental well-being is paramount in leading a quality and productive life. Exposure to multiple stressors in their daily work has highlighted the mental well-being crisis among health professionals as a priority in addressing attrition, dissatisfaction, and reduced productivity [1,2]. Numerous studies have expounded mental well-being afflictions, such as anxiety, depression, stress, and burnout, measured by diverse assessment tools, including the Depression Anxiety Stress Scales-21 (DASS21) and the Mayo Clinic Well-Being Index (MWBI) [3,4,5]. These studies imply a correlation between mental well-being issues and adverse workplace encounters, as well as diminished professional efficacy, increased absenteeism, and instances of job-related leave. During the COVID-19 pandemic, physicians encountered substantial challenges while discharging their professional responsibilities in exceptionally demanding and stressful conditions. These challenges rendered them particularly vulnerable to occupational hazards, adverse events, and secondary victimization, thus necessitating professionals’ urgent and meticulous consideration and attention [6,7] and underscoring the importance of monitoring and timely support from organizational and labor systems to preserve their mental well-being.

In the context of Serbia, there exists a dearth of scientific research pertaining to the mental well-being of healthcare professionals. The available evidence suggests that younger clinicians with limited experience, unprepared for change, have faced challenges in adapting to their new responsibilities during the pandemic [8]. Amid the pandemic, it became customary to reallocate healthcare personnel to different units within the healthcare system. Nevertheless, certain supervisors resorted to verbal threats of termination should personnel resist such reassignments [9]. Emotional exhaustion as a symptom of burnout was reported, followed by moderate compassion fatigue and a lower level of self-efficiency among health workers during the pandemic [10]. The fact that the frequency of poor mental well-being among health workers remains unexplored in Serbia underscores the imperative for further studies to be undertaken to safeguard their occupational efficacy and resilience and, in turn, patient safety.

In managing pandemics and other health emergencies, environmental disasters, and public health crises, the indispensable position of primary care is undeniable. General practitioners (GPs) are not only the first point of contact for potentially infectious patients but are also involved in all phases of the epidemic response [11]. Their role in providing health education services, screening, and medical surveillance and treating and monitoring patients with symptoms and consequences is crucial in preventing and reducing viral transmission in a society hit by an epidemic or a pandemic [12]. At the same time, they must maintain continuity and comprehensiveness of primary health care for all patients in need, leaving no one behind. Consequently, the GP practice has changed to include additional responsibilities, the adoption of digital technology, new administrative services, and face-to-face online consultations while at the same time managing work overload, often in the conditions of insufficient resources, time strains, and redeployed co-workers [5,13]. During pandemics, GPs face significant challenges, including increased exposure to sources of infections, fear of disease transmission to family members, and the need for physical isolation with less opportunity for empathetic social exchange. Lack of training and support from authorities could contribute to medical errors, which negatively affect the mental well-being of GPs [4,7]. As healthcare professionals, researchers, policymakers, and organizations involved in healthcare management and support, it is important to acknowledge these challenges and work toward providing the necessary support. Experiences from previous viral outbreaks indicate potential long-term and persistent mental well-being problems after the crises have ended [14]. Furthermore, they underscore the usefulness of timely assessments of the mental well-being statuses of frontline healthcare workers, necessitating culturally tailored and validated assessment tools.

Research on the adverse psychological impact of the COVID-19 pandemic on GPs is growing, pointing out the timely recognition of mental well-being deterioration of GPs in emergencies as a priority for all—patients, GPs, and managers in the health system. Timely recognition of the decline of GPs’ mental well-being is needed to avoid potentially devastating consequences on their health and work performance, such as abuse of drugs and alcohol and suicidal ideation among GPs [7], absenteeism, low commitment to work, and increased turnover [5], as well as impairment of the quality of health care, patient safety, and satisfaction [6] jeopardizing the overall primary healthcare system’s performance and sustainability [5]. The MWBI and its correlation with the DASS21 are crucial elements that healthcare professionals, researchers, policymakers, and organizations involved in healthcare management and support should thoroughly understand. If there is a strong correlation, the MWBI could be used to quickly assess the well-being of GPs working in crises, an area where its effectiveness has not been explored; provide valid actionable information; and enable the development of tailored mental well-being.

This study was conducted to assess the mental well-being of general practitioners during the COVID-19 pandemic. Our initial focus was on evaluating the appropriateness of employing the MWBI for Serbian GPs. Our secondary objective was to investigate the link between GPs’ MWBI scores and their personal and job-related characteristics.

## 2. Materials and Methods

### 2.1. Study Design and Setting

A cross-sectional approach was used to study the mental well-being of GPs in Serbia within the international project “Primary Health Care in times of COVID-19” (PRICOV-19), initiated and coordinated by Ghent University (Belgium). The questionnaire developed to assess how GPs deliver safe, efficient, effective, and fair care during the pandemic was validated and piloted among 159 GP practices in Flanders (Belgium) [15]. The final version of the questionnaire in English involved GPs self-assessing the impact of the COVID-19 pandemic on their roles, responsibilities, and well-being, as well as how GP practices were organized and responded to and adapted to crises in 38 countries (including 37 European countries and Israel).

This study constitutes a pilot project in which the Serbian research team undertook the translation of the survey into Serbian using the forward–backward method. Furthermore, we adapted the survey to align with the cultural context, incorporating considerations such as the organizational structure of primary healthcare and job descriptions. Data collection from GPs’ practices in Serbia was conducted between and during the peaks of the COVID-19 pandemic, from January to June 2021.

### 2.2. Study Participants and Variables

The population of interest for this study comprised licensed and active GPs in Serbia in 2021. According to the routine statistics, in a total of 141 primary healthcare centers, there were 3523 medical doctors—general practitioners, in addition to 1381 GP specialists and 235 medical doctors in GP training specialization [16]. For the purpose of GP sampling in this study, we adhered to the predetermined recruitment procedure specified in the pre-published PRICOV-19 protocol [15]. Our priority was to secure a sample that encompassed a sample representation of typical GPs, thereby mitigating selection bias based on their personal and job-related characteristics. The sufficient sample size for assessing the frequency of MWBI scores of ≥2 among general practitioners with a precision of 0.1, a confidence level of 0.95, and an assumed frequency of the phenomenon under investigation of 57% (according to pre-published data from Cholewa, 2023) [17] is 95 respondents.

The questionnaire with consent for participation was distributed in two stages. Initially, it was disseminated anonymously online using the email database of the Serbian Medical Chamber. However, due to irregular updates to the email list, we were advised to personally contact individuals for whom email delivery failed. Consequently, in the second stage, the research team approached GPs in primary healthcare centers to assist in distributing the questionnaire in paper format through their professional connections. Out of the 130 returned questionnaires, a total of 116 GPs completed the questionnaire voluntarily (89%). The sample size of this study aligns with the range of samples from the PRICOV-19 consortium countries, which spans from 13 in Malta to 370 in Belgium [15]. It is pertinent to emphasize that during this phase of the pandemic, the majority of countries were constrained to employ a convenience sampling approach [15]. The anonymized data have been uploaded to the Research Electronic Data Capture (REDCap) tool hosted at Ghent University to securely store the self-reported data regarding GPs’ personal and job-related characteristics as well as the key outcome variable of interest: the mental well-being of GPs [18].

The personal characteristics of a general practitioner were the following: gender (male or female), age (continuous variable, years), work experience (continuous variable, years), marital status (dichotomous variable, married or not married, with the latter including individuals who have never been married, widows/widowers, and divorced persons), and socioeconomic status (poor, average, and good or very good compared with the average for Serbia). Socioeconomic status encompassed the total household income generated by all members, including both the unemployed and employed.

The job-related characteristics of a GP’s practice included the practice’s location (urban, such as a big city; non-urban, such as a suburb or small town; mixed urban–rural, or rural) and the patient composition in the GP’s practice, including patients with COVID-19 (yes or no), the workload with patients with chronic conditions (above or below the average volume for the GP’s practice), and the workload with patients aged 70 years and older (above or below the average volume for the GP’s practice). When referring to “below average”, it includes responses such as “not above average”, “approximately the average”, and “I do not know”. Furthermore, the GPs’ opinions were gathered regarding their tasks during the COVID-19 pandemic about increased responsibilities, preparedness for task shifting, training needs for amended responsibilities, and adequate time in their agendas for reviewing new guidelines or going through the relevant and reliable scientific literature. The GPs responded to these questions on a five-point Likert scale (strongly disagree, disagree, neutral, agree, strongly agree). For modeling purposes, the GPs’ responses were transformed into dichotomous responses: “agree” (encompasses “agree” and “strongly agree”) and “disagree”, which includes all other answers.

The outcome variable, the GPs’ mental well-being, was assessed in two ways: the expanded nine-item scale of the MWBI and the DASS21—the official standardized Serbian version. Within the PRICOV-19 study, we obtained permission to use the original MWBI.

The MWBI instrument consisted of nine items. Seven items assessed the presence (“yes” or “no”) of psychological distress, including burnout, depression, fatigue, stress, and quality of life. The remaining two items evaluated the respondents’ agreement on work relevance and their satisfaction with work–life balance. The responses were given on a seven-point Likert scale for work relevance (ranging from “strongly disagree” to “strongly agree”) and a five-point Likert scale for work–life balance satisfaction (ranging from “strongly disagree” to “strongly agree”). The MWBI was scored as follows: One point was assigned for each “yes” answer for the first seven items. For the last two items, one point was added for the response options “strongly agree” and “agree,” and one point was subtracted for the response options “strongly disagree” and “disagree”. No adjustment was made for neutral response options. Therefore, the total MWBI score ranged from −2 to 9, where higher scores indicated poorer mental well-being. Previous studies have suggested that the threshold MWBI score to identify GPs with a higher risk of psychological distress is 2 [4].

The DASS21 instrument was used to identify the main symptoms of depression, anxiety, and stress, as well as the general psychological distress among GPs. The DASS21 comprises three subscales for depression, anxiety, and stress, each containing seven items. Participants used a 4-point scale to rate their symptoms of psychological distress over the past week, from 0 (never) to 3 (mostly or almost always). The scores for depression (DASS21 Depression), anxiety (DASS21 Anxiety), and stress (DASS21 Stress) are calculated by adding up the scores for the relevant items and then multiplying that sum by 2. Based on the DASS manual [19], the general psychological distress was measured by applying the following threshold values: a total DASS21 score of >25, a DASS21 Depression score of >9, a DASS21 Anxiety score of >7, and a DASS21 Stress score of >14.

### 2.3. Statistical Analysis

Basic descriptive statistics were utilized to summarize the sample characteristics of the GPs. Means, standard deviations, medians, minimums, and maximums were used to present the continuous and scale variables. In contrast, the categorical ones were presented with absolute numbers and percentages.

To assess the correlation between the MWBI scores and DASS21 scores, we applied Spearman’s correlation coefficient, indicating a strong correlation if it was ≥0.7 [20]. Linear regression analysis was used to identify a significant univariate and multivariate association between the outcome variable (MWBI score) and independent variables (personal and job-related characteristics). All variables with statistical significance at the 0.05 level in the univariate analysis were included in the multivariate analysis using the “Enter” method. The variance inflation factor (VIF) did not reveal any multicollinearity among the factors in the regression model. The analyses were performed by both IBM SPSS Statistics 22 (IBM Corporation, Armonk, NY, USA) and R-4.0.0 software (R Core Team, 2020).

## 3. Results

The main personal and job-related characteristics of the 116 GP respondents are summarized in Table 1. The mean responder’s age was 44.7 years, while the median of the years of practice was 11. Two-thirds of the respondents were females and married, while half had good socioeconomic statuses. Almost all respondents worked with COVID-19 patients. About half of the survey participants indicated that the proportion of their patient population with chronic conditions and aged 70 years and above in their practice was higher than the national average. Though most respondents claimed their responsibility had increased since the COVID-19 pandemic, slightly more than a fifth agreed that they were unprepared for task shifting, and a third believed they needed additional training. Three-fifths of respondents indicated they lacked sufficient time to review new guidelines or engage with the pertinent literature during the pandemic.

In total, 101 GPs completed both the MWBI and DASS21 questionnaires (Table 2). The mean MWBI score among the respondents was 3.3 (SD 2.7); it ranged from −2 to 9, with a median of 3. The median DASS21 Total, Depression, Anxiety, and Stress scores were 24 (0, 122), 6 (0, 42), 6 (0, 40), and 14 (0, 40), respectively.

**Figure 1:**
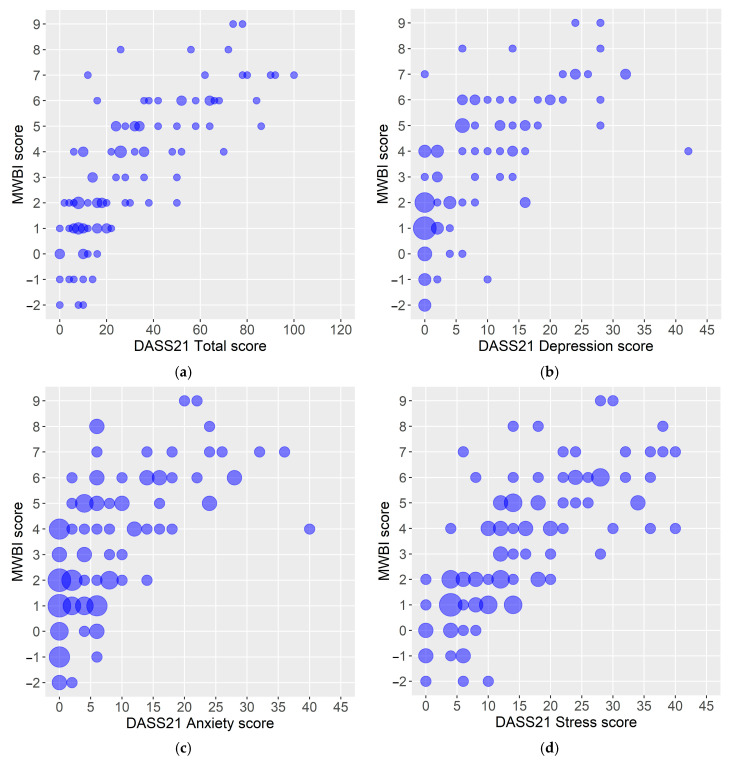
(**a**–**d**) Scatter plots of respondents’ MWBI scores and DASS21 scores: (**a**) MWBI score vs. DASS21 Total score, (**b**) MWBI score vs. DASS21 Depression score, (**c**) MWBI score vs. DASS21 Anxiety score, and (**d**) MWBI vs. DASS21 Stress score. Legend: MWBI—Mayo Clinic Well-Being Index; DASS21—Depression Anxiety Stress Scales-21. Note: The circle size depends on the number of respondents with the same score value.

A more robust and positive linear association was illustrated between the DASS21 score for the stress dimension and the MWBI score than between the anxiety and depression dimensions of the DASS21 score and the MWBI score (Figure 1).

The Spearman correlation coefficient values confirm a strong, positive, and significant correlation between the MWBI and DASS21 scores (Table 3). In total, 71.3% of respondents had MWBI scores of ≥2. Almost half of the respondents (48.5%) had total DASS21 scores > 25.

Multivariate linear regression revealed two potential independent predictors of poorer mental well-being among GPs (Table 4). With every decrease in socioeconomic status (from very good to poor), the MWBI score increased by 0.893. Inadequate time in the GPs’ agenda(s) for reviewing new guidelines or going through the relevant and reliable scientific literature (B = −1.137; *p* = 0.033) was independently and significantly related to higher MWBI scores (i.e., deterioration in mental well-being). No multicollinearity between the potential predictors was detected (the VIFs for the independent variables in the model ranged from 1.07 to 1.32). The whole model is statistically significant (*p* < 0.001) and explains 21% of the variation in the dependent variable.

## 4. Discussion

In times of emergency, primary healthcare organizations must monitor the mental well-being of GPs to identify those who may need mental well-being support. This allows for the implementation of activities to promote mental well-being and ensure appropriate GP service provision. Our pilot study focused on the appropriateness of utilizing the MWBI for GPs during periods of crisis and the link between the MWBI scores of GPs and their personal and job-related characteristics. The assessment findings show a strong correlation between the MWBI score, the total DASS21 score, and the separate DASS21 scores for the depression, anxiety, and stress dimensions. In a similar study, the correlation between the MWBI and DASS21 among medical students was moderate, probably because of the contextual and respondents’ characteristics [21]. The MWBI was initially developed to screen for common manifestations of stress (i.e., depression, burnout, fatigue, anxiety, stress, and mental quality of life) among medical students [21,22,23]. Later, it was modified and validated for use among healthcare professionals [24,25,26,27] and the general US working population [28]. Our analysis indicates the MWBI’s pronounced efficiency in GP practices necessitating prompt action during the COVID-19 pandemic. The MWBI’s advantage lies in its simplified application process, resulting from its concise nine-item structure, and the bifurcation of items into personal and job-related dimensions. Nonetheless, this delineation restricts MWBI efficacy in screening mental well-being issues, confining its utility to preliminary assessment purposes. It is advisable for primary care physicians and practice managers to actively monitor the MWBI scores of their personnel and recognize indicators signaling the necessity to arrange for professional support for individuals exhibiting declines in mental well-being.

In our research, more than two-thirds of GP respondents exhibited high susceptibility to psychological distress. This figure closely aligns with the percentage observed among Slovenian family physicians (68.5%) and exceeds that of Belgian GPs (57%) [17,29] and 65% of GPs across 33 European countries who reported high MWBI scores in the PRICOV-19 project [4]. The mean ± standard deviation of the MWBI score in Slovenia was determined to be 3.3 ± 2.6 [29], which coincided with the findings in our study. The PRICOV-19 study identified a lower mean MWBI score of 2.7 ± 2.7 [17]. Against the primary healthcare workforce in Serbia, before the pandemic, about three percent of the adult population had been diagnosed with chronic depression [30]. The prevalence of mental disorders during the 2021 pandemic year was not significantly impacted by the frequently reported stressors related to COVID-19 [31]. Similarly, about four percent of the reasons for the primary healthcare visits were due to mental health disorders over the pandemic period [16]. Medical students with inadequate knowledge of COVID-19 exhibited a heightened propensity for depression and anxiety in response to the ongoing outbreak [32]. This observation implies potential mental well-being vulnerability among medical personnel unprepared for their new roles and responsibilities within the pandemic context.

As in the PRICOV-19 study [4], we also found an association between insufficient time for GPs to review new guidelines or the relevant literature and worsening mental well-being, which correlates with poor MWBI scores. However, other studies indicated that less experienced GPs [4,29] and those reporting inadequate workload distribution exhibited higher distress scores [29]. This pattern was particularly notable among those serving more vulnerable patient populations, those tending to patients facing financial difficulties, and those operating in single or duo practices [4]. Our research endorses the findings that allocating sufficient protected time in a GP’s schedule to review guidelines or engage with the scientific literature functions as a crucial protective factor for mental well-being.

### 4.1. Strengths and Limitations

The main strength of this study is that we both identified the cut-off value of the MWBI score pointing at the presence of the risk of distress and demonstrated the utility of monitoring the increases in distress risks via providing evidence of the distribution of the continuous MWBI scores among GPs working at the frontline in a pandemic. It is worth noting that cut-off values are often derived from empirical studies conducted in diverse contexts and cultures. For instance, in Belgium [30], a MWBI threshold of 2 was established. In contrast, in the United States [24], a threshold score of 4 or higher was deemed an appropriate initial point for identifying physicians with low MWBI scores, high fatigue, or recent suicidal ideation. However, a score of 3 or above was a significant threshold for identifying dentists at an elevated risk of distress [27]. Continued culturally specific attitudes toward mental well-being issues may contribute to the varying strengths of correlation observed. For instance, a moderate correlation was found in Malaysia [21], while we found a strong correlation between the MWBI and DASS21 subscales. The strength of our study lies in using a culturally validated tool for collecting MWBI data in the Serbian GP context. This tool can also compare GPs across Europe, such as in the PRICOV-19 study. Additionally, it allows for tracking changes in GPs’ MWBI scores over time and addressing unfavorable changes at an organizational level.

This study has limitations due to the small, non-randomized sample obtained between and during the peaks of the COVID-19 pandemic in the first half of 2021. Nevertheless, the sufficient number of the sample size for assessing the frequency of MWBI scores of ≥2 among GPs with a precision of 0.1, a confidence level of 0.95, and an assumed frequency of the phenomenon under investigation of 57% (according to pre-published data from Cholewa, 2023 [17]) is 95 respondents. Furthermore, the personal characteristics of the GPs in our sample resemble the age and sex profiles of medical doctors in Serbia (approximately 25% of all medical doctors are GPs), characterized by a middle-aged workforce and a predominance of females [16]. Due to stringent public restrictions, the researchers encountered challenges in physically disseminating the questionnaires to the GPs’ practices and achieving a more comprehensive and randomized response rate within the study deadline. In light of the concerning reports regarding the “parallel pandemic” affecting health workers [33] and possible under-registration of related mortality (e.g., ischemic heart syndrome, COVID-19, and suicides) [34], it is imperative to strategically plan a comprehensive study of the MWBI to mitigate the risk of mental disorders within this demographic. Our findings could serve as a fundamental reference point for this initiative.

Additionally, the DASS21 questionnaire took a long time to complete, resulting in fewer completed forms than the MWBI questionnaire. Nevertheless, the findings from this study indicate the statistical significance of these study models. It is essential to acknowledge the potential impact of the unaccounted variables in future studies, such as the number of dependents, household income, or other pertinent factors not included in the current survey. These factors should be considered to enhance the model’s explanatory power. Despite the evident utility of the MWBI instrument demonstrated in numerous empirical studies, further research covering larger samples is needed to establish its generalizability and relevance for the entire GP population. This cross-sectional study’s design precluded the establishment of causal relationships and instead focused on examining the correlations regarding the mental well-being of GPs in crises. These study findings underscored the imperative for managerial attention to safeguard the mental well-being of the GP workforce.

### 4.2. The Policy, Practical, and Scientific Implications of This Study

This study builds upon the foundational work of the PRICOV-19 Study and provides significant insights into the well-being of GPs in Serbia during the pandemic. Notably, this study is the first ever of its kind in Serbia, even though the country has previously confronted the smallpox and Swine Flu outbreaks, among other emergency situations, during which GPs were perpetually on call. In that sense, this study brings new evidence for addressing the context-specific needs of GPs during crises. This study presents valuable insights that may contribute to policy and practical applications and scientific considerations. The policy initiatives to enhance the well-being of GPs and that can benefit both providers and patients involve creating mental health support systems into policies and promoting work–life balance and self-care. In addition, creating job descriptions that allow sufficient time for communicating and appraising new clinical evidence within institutional emergency preparedness and response plans could have a protective effect on mental well-being. Respite care for general practitioners is also recommended as a form of short-term relief, allowing them time to rest and recharge. In a practical sense, operational changes should aim at implementing mental well-being screening and monitoring for GPs and evaluating workload and job demands in emergencies.

From the scientific standpoint, this robust study’s design and findings highlight the critical requirement to support the well-being of GPs during the challenging times of the COVID-19 pandemic. At the time of outbreak, the World Health Organization drew a parallel between COVID-19 and the devastating smallpox outbreak, which resulted in approximately 300 million deaths in the 20th century [35]. By knowing that, we should appreciate the significance of understanding the gravity of the GPs’ situation during crises, as our study has illustrated. These study findings call for collaborative work to enhance resilience and support systems for GPs in the face of such unprecedented challenges.

Our study draws on original data and uses two powerful and standardized tools to enhance our understanding of the similarities and diversities of the mental well-being of general practitioners across Europe. Moreover, the selection of the MWBI and the DASS-21 is supported by their prevalence in the literature. Over ten years ago, the Mayo Clinic Index was validated on more than 25,000 individuals in many professions and job types and its ability to predict a variety of different occupational and personal outcomes was evaluated [28]. It is a tool that supports accurate clinicians’ well-being measurement. The commonly used surveys, such as the DASS-21, are longer and cumbersome and do not provide the functionality needed to adequately measure and support health staff needs in critical times. Since the DASS-21 has been proven to credibly assess the emotional states of depression, anxiety, and stress across numerous countries [36,37] and in Serbia [38], it was necessary to verify the MWBI utility for the same purpose.

This study contributes to scientific knowledge by exploring lesser-researched areas in a specific context, Serbian GPs, and thoughtfully examining prevailing viewpoints to provide solid scientific support. Given that GP mental well-being courses during the peaks of pandemics are a phenomenon and a natural experiment worth exploring, the implications of our studies encourage us to broaden our management expertise and explore new areas beyond our current understanding of clinical practice.

## 5. Conclusions

The research conducted in Serbia indicates that the MWBI is a valid and reliable tool for promptly assessing the mental well-being of GPs during times of crisis. Furthermore, lower socioeconomic statuses and insufficient time in the GPs’ schedules for reviewing new guidelines or engaging with the relevant scientific literature are contributing factors to the diminished mental well-being of Serbian GPs. This study also underscores the significance of addressing the characteristics of GPs’ practices in conjunction with personal traits, emphasizing that the structures and context of the primary healthcare system play a crucial role in safeguarding the mental well-being of GPs.

General practitioners and practice managers can utilize the MWBI, which consists of nine simple questions and six straightforward dimensions, to assess individual and organizational well-being as needed, all within a brief time frame of under one minute. This approach enables comprehensive tracking and reporting of potential distress risks and ensures GP staff can access necessary support resources. This study shows that the MWBI offers an accuracy level comparable to the DASS-21, promoting a proactive approach to well-being within the clinical practice.

## Figures and Tables

**Table 1:** Personal and job-related characteristics of the general practitioners (*n* = 116) during the COVID-19 pandemic.

Personal Characteristics of a General Practitioner		
Gender, *n* (%)	Male	25 (21.6)
	Female	91 (78.4)
Age, years	mean (SD)	44.7 (11.1)
Work experience, years	median (min, max)	11.0 (0.5, 39.8)
Marital status, *n* (%)	Married	88 (75.9)
	Not married	28 (24.1)
Socioeconomic status, *n* (%)	Poor	6 (5.2)
	Average	42 (36.2)
	Good	60 (51.7)
	Very good	8 (6.9)
**Job-Related Characteristics of General Practitioner’s Practice, *n* (%)**		
Location of practice	Urban	61 (52.6)
	Not urban	55 (47.4)
Working with COVID-19 patients	Yes	107 (92.2)
	No	9 (7.8)
Workload of patients with chronic conditions	Above average	53 (47.3)
	Below average	59 (52.7)
Workload of patients aged 70 years and older	Above average	51 (55.7)
	Below average	64 (44.3)
Increased responsibilities	Agree	101 (87.1)
	Disagree	15 (12.9)
Unprepared for task shifting	Agree	26 (22.4)
	Disagree	90 (77.6)
Training is needed for the amended responsibilities	Agree	38 (32.8)
	Disagree	78 (67.2)
Adequate time for reviewing new guidelines	Agree	43 (40.2)
	Disagree	64 (59.8)

Legend: *n*—number of respondents; SD—standard deviation.

**Table 2:** The MWBI and DASS21 scores of the general practitioners (*n* = 101).

Score	Mean (Standard Deviation)	Median (Minimum; Maximum)
MWBI ^a^	3.3 (2.7)	3 (−2; 9)
DASS21 Total ^b^	31.7 (27.0)	24 (0; 122)
DASS21 Depression ^c^	8.3 (9.6)	6 (0; 42)
DASS21 Anxiety ^d^	8.1 (9.0)	6 (0; 40)
DASS21 Stress ^e^	15.3 (10.7)	14 (0; 40)

Legend: MWBI—Mayo Clinic Well-Being Index; DASS21—Depression Anxiety Stress Scales-21. ^a^ For dichotomous MWBI items 1–7, the Kuder–Richardson (KR20) coefficient was 0,72. ^b^ The Cronbach alpha for the DASS21 Total score was 0.96. ^c^ The Cronbach alpha for the DASS21 Depression score was 0.93. ^d^ The Cronbach alpha for the DASS21 Anxiety score was 0.90. ^e^ The Cronbach alpha for the DASS21 Stress score was 0.92.

**Table 3:** Correlation between the general practitioners’ MWBI and DASS21 scores (*n* = 101).

Spearman’s Correlation Coefficient Sig. (Two-Tailed)	DASS21 Total	DASS21 Depression	DASS21 Anxiety	DASS21 Stress
DASS21 Depression	0.905			
	<0.001			
DASS21 Anxiety	0.863	0.714		
	<0.001	<0.001		
DASS21 Stress	0.946	0.804	0.725	
	<0.001	<0.001	<0.001	
MWBI	0.791	0.730	0.660	0.759
	<0.001	<0.001	<0.001	<0.001

Legend: MWBI—Mayo Clinic Well-Being Index; DASS21—Depression Anxiety Stress Scales-21.

**Table 4:** Potential predictors of poor mental well-being (higher scores of the Mayo Clinic Well-Being Index) for general practitioners during the COVID-19 pandemic (*n* = 101).

Variables	Linear Regression			
	Univariate		Multivariate	
	Unstandardized B	*p*	Unstandardized B	*p*
**Personal characteristics of a general practitioner**				
Gender (female vs. male)	−0.321	0.608		
Age (years)	−0.004	0.878		
Work experience (years)	0.005	0.808		
Marital status (married vs. not married)	0.607	0.317		
Socioeconomic status (level)	−1.090	**0.003**	−0.893	**0.021**
**Job-related characteristics of general practitioner’s practice**				
Working with COVID-19 patients (yes vs. no)	−0.085	0.932		
Location of practice (not urban vs. urban)	−0.775	0.143		
Workload of patients aged 70 years and older (above average vs. below average)	0.972	0.068		
Workload of patients with chronic conditions (above average vs. below average)	1.306	**0.013**	0.971	0.057
Increased responsibilities in the practice (agree vs. disagree)	0.429	0.589		
Unprepared for task shifting (agree vs. disagree)	1.654	**0.009**	0.507	0.460
Training is needed for the amended responsibilities (agree vs. disagree)	1.119	**0.043**	0.200	0.736
Adequate time for reviewing new guidelines (agree vs. disagree)	−1.578	**0.004**	−1.137	**0.033**

Note: Significant findings where *p* < 0.05 are marked in bold.

## Data Availability

All data and materials used in this study are available upon reasonable request.
